# Supplementary material for: Using deep learning-based artificial intelligence electronic images in improving middle school teachers’ literacy
Source: PeerJ Comput Sci. 2024 Mar 29;10:e1844. doi: 10.7717/peerj-cs.1844 (PMC11041997; doi:10.7717/peerj-cs.1844)
Supplement: Supplemental Information 2 [file peerj-cs-10-1844-s002.zip › Code Description and Configuration.docx]

**Code description:**

1. **Import necessary libraries and modules:** Firstly, some necessary Python libraries were imported, including NumPy, neural network modules in PyTorch, optimizer, and model and image transformation modules in Torchvision. Moreover, a custom data loader called DataLoader is also imported, which loads electronic image data during the teacher's teaching process.
2. **Define the Teacher Intelligence Model:** A PyTorch model class called the Teacher Intelligence Model is defined in this part. The model uses a pre-trained SOLO classification model as a feature extractor and replaces its final classification layer with a new linear layer suitable for teachers' intelligent classification tasks. The model has a forward method for performing a forward propagation process, which includes SOLO feature extraction and teacher intelligence classification.
3. **Create a model instance:** Here, an instance of the Teacher Intelligence Model class is created, and the number of categories to classify is specified.
4. **Define the loss function and optimizer:** The loss function (cross-entropy loss) and optimizer (Adam optimizer) used to train the model are defined. These will calculate the loss and update the model's weights during training.
5. **Load electronic image data for teacher teaching:** The custom data loader DataLoader is used to load electronic image data for training. This data loader may require further configuration, specifying the dataset's path, batch size, and whether to shuffle the data.
6. **Training process:** In this part, the model starts training. It iterates through batch images and labels in the data loader, loads data into the GPU (if available), performs forward propagation, backpropagation, calculates losses, and updates model parameters. Training information is output at the end of each cycle, including the current period and loss value.
7. **Evaluate the model:** After the model training ends, the code switches to evaluation mode and evaluates the model using the test dataset. This part loads the test data, performs forward propagation to get the model's predictions, and calculates accuracy and other evaluation metrics.

**Configuration:**

Parameter configuration and settings:

- num_classes: The number of categories to classify is specified and set according to your question.
- learning_rate: The learning rate of model training is specified.
- dataset_path: The dataset path for training and testing is specified.
- batch_size: The batch size is specified during training and testing.
- num_epochs: The number of epochs of training is specified.
- device: The device the model runs on is specified, such as CPU or GPU.

In addition, you need to provide a dataset that fits the problem and ensure that the labels of the dataset match the number of categories that the model expects.

**How to use the algorithm:**

If you want to use this code, you can follow these steps:

Parameters in the code are configured, covering num_classes, learning_rate, dataset_path, batch_size, num_epochs, etc.

You need to prepare datasets that apply to your problem, ensuring that they are under the specified dataset path and that the labels of the datasets match the num_classes.

The code is run to create model instances, define loss functions and optimizers, and load data.

The training process is performed, and the model is trained on the training data.

After the training, the model evaluation process is performed. The model is evaluated using a test dataset, and performance metrics are calculated.
